# Impact of Tamm plasmon structures on fluorescence and optical nonlinearity of graphene quantum dots

**DOI:** 10.1038/s41598-024-62694-9

**Published:** 2024-06-10

**Authors:** Hasana Jahan Elamkulavan, Nikhil Puthiya Purayil, Sanjay Subramaniam, Chandrasekharan Keloth

**Affiliations:** Laser and Nonlinear Optics Laboratory, Department of Physics, National Institute of Technology, Calicut, 673601 India

**Keywords:** Graphene quantum dots, Tamm plasmon, Photonic crystal, Optical nonlinearity, Fluorescence, Optical limiting, Optics and photonics, Physics

## Abstract

Graphene Quantum Dots (GQDs) are crucial in biomedicine for sensitive biosensing and high-resolution bioimaging and in photonics for their nonlinear optical properties. Integrating GQDs with photonic structures enhances optical properties by optimizing light-matter interactions and enabling precise control over their emission wavelengths. In this work, we explore a facile synthesis method for GQDs by pulsed laser irradiation in chlorobenzene and highlight the transformative potential of Tamm Plasmon Cavity (TPC) structures for tuning and amplifying the photoluminescence and nonlinear optical properties of GQDs. The characterization of GQDs revealed their exceptional properties, including efficient optical limiting and stable photoluminescence. The study demonstrated that the TPC structure significantly amplifies nonlinear optical effects due to the high light-matter interaction, indicating the potential for advanced optical systems, including optical limiters and nonlinear optical devices. Furthermore, introducing GQDs into the TPC structure leads to a significant enhancement and tuning of fluorescence emission. The Purcell effect, in combination with the confined electromagnetic fields within the TPC, increases the spontaneous emission rate of GQDs and subsequently enhances the fluorescence intensity. This enhanced and tunable fluorescence has exciting implications for high-sensitivity applications such as biosensing and single-molecule detection.

## Introduction

In the rapidly developing fields of photonics and nanotechnology, scientists and researchers are looking for new ways to control and utilize the unique qualities of light. The search for compact, efficient, and flexible optical devices has sparked a revolution in photonics research. Traditional plasmonic systems have been extensively studied and employed for their capacity to concentrate electromagnetic fields at metal-dielectric interfaces^1–3^. However, these structures often have limitations such as high energy losses and restricted tunability. Tamm plasmon cavity structures have been developed, providing an innovative solution to these challenges by incorporating distributed Bragg reflectors (DBRs) into the architecture^4^. This ingenious design not only reduces energy losses but also enables precise control over resonance wavelengths, making TPCs exceptionally versatile and adaptable^5^.

Tamm plasmons (TPs), introduced by Kaliteevski et al*.* in 2007^6^ and later explored experimentally by M.A. Sasin and his team in 2008^7^, combine the advantages of plasmonics with the precision of DBRs. In 2017, Symonds et al*.* demonstrated the emergence of a "super Tamm plasmon" resonance characterized by a high-quality factor^8^. This exceptional resonance was achieved by skillfully adjusting the interaction between the metal layer and the DBR by incorporating a dielectric spacer layer. Recently, TPs were used to direct and enhance the emission of nanomaterials^9–11^. Furthermore, TPs have exhibited remarkable potential in the realm of optical nonlinearity^12^. Hence, they are being considered for innovative applications in perfect absorbers^13^, nanoscale lasers^14^, filters^15^, bistable switches^16^, thermal emitters^17^, solar photovoltaic cells^18^, photodetectors^19^, and many other applications^4^. TPCs offer a transformative platform for manipulating light-matter interactions and introducing nanoscale confinement that can significantly amplify electromagnetic fields. However, their true potential emerges when paired with nanomaterials, the versatile building blocks of the nanotechnology era.

In the space where nanomaterials meet the fascinating world of optics, one class of nanocrystals stands out for its extraordinary versatility and appealing attributes^20^: graphene quantum dots. These nanoscale wonders, composed of a few layers of graphene sheets with lateral dimensions typically less than 10 nm, have ignited strong interest among researchers and scientists owing to their unique capabilities^21^. They offer precise size control, exhibit quantum confinement effects, and shows strong photoluminescence, making them valuable for applications such as bioimaging and sensing^22,23^. GQDs are biocompatible, highly photostable, and can be modified with functional groups, enhancing their versatility for diverse applications^22,24^. These tiny luminophores possess an innate ability to emit intense, tunable fluorescence, making them promising for use in the field of nanophotonics and imaging^25^.

The fluorescence manipulation of GQDs has been an area of immense research worldwide in recent decades. Their role extends to materials science, environmental monitoring, security, and fundamental research, underlining their versatile and multifaceted significance in addressing a wide array of scientific, technological, and societal challenges^26^. Enhancing the quantum yield and reducing background noise are essential for overcoming limitations in the sensitivity and selectivity of fluorescence-based sensors and other devices, ultimately improving their performance.

In addition to their inherent fluorescence, GQDs also exhibit fascinating nonlinear optical properties^27,28^. The enhancement of optical nonlinearity in GQDs is of great importance for many advanced photonics and materials science applications^29^. By manipulating the nonlinear optical properties of GQDs, we unlock the potential for high-efficiency photonic devices such as ultrafast optical switches, frequency converters, and all-optical signal processors. The ability of GQDs to exhibit nonlinear effects such as two-photon absorption and Kerr nonlinearity not only accelerates data processing and telecommunications but also enables the development of quantum technologies where efficient nonlinear optical materials are in high demand^30,31^.

There are different methods adopted for the synthesis of GQDs. Top-down methods, such as oxidative cleavage^32^, hydrothermal^33^ or solvothermal^34^ methods, ultrasonic-assisted^35^ or microwave-assisted processes^36^, and electrochemical oxidation^37^, involve the mechanical or chemical breakdown of larger graphene sheets into smaller pieces, resulting in GQDs^38^. Bottom-up methods, including molecular carbonization methods and other methods, involve the self-assembly of smaller molecules into GQDs through dehydration and carbonization or other chemical reactions^39,40^. One such bottom-up methods which has gained attention in recent years is the pulsed laser irradiation in liquid (PLIL) technique^41^, in particular, PLIL in benzene derivatives. Shiju et al*.*^42^ and Nancy et al*.*^30^ demonstrated the synthesis of GQDs by pulsed laser irradiation in toluene, which is a commonly used benzene derivative. Despite the various methods available, there are still challenges to be addressed, such as scalability, control over size and shape, purification, stability, and cost-effectiveness. To overcome these challenges, researchers are exploring new methods and techniques for synthesizing GQDs.

In this work, we discuss a facile synthesis method and the properties of GQDs, showing their significance for both fundamental research and practical applications. We then explored the design principle of TPC structures to revolutionize the fluorescence and optical nonlinearity of GQDs, offering exciting prospects for applications in diverse fields ranging from biosensing to telecommunications. To the best of our knowledge, this is the first paper reporting the synthesis of GQDs by laser irradiation in chlorobenzene, which is also a benzene derivative solvent, and tuning its fluorescence and nonlinear properties together with a photonic structure (Tamm cavity structure) composed of a minimal number of bilayers of spin-coated silicon dioxide (SiO_2_) and titanium dioxide (TiO_2_) as DBR materials.

## Methods

### Synthesis

**Figure 1:**
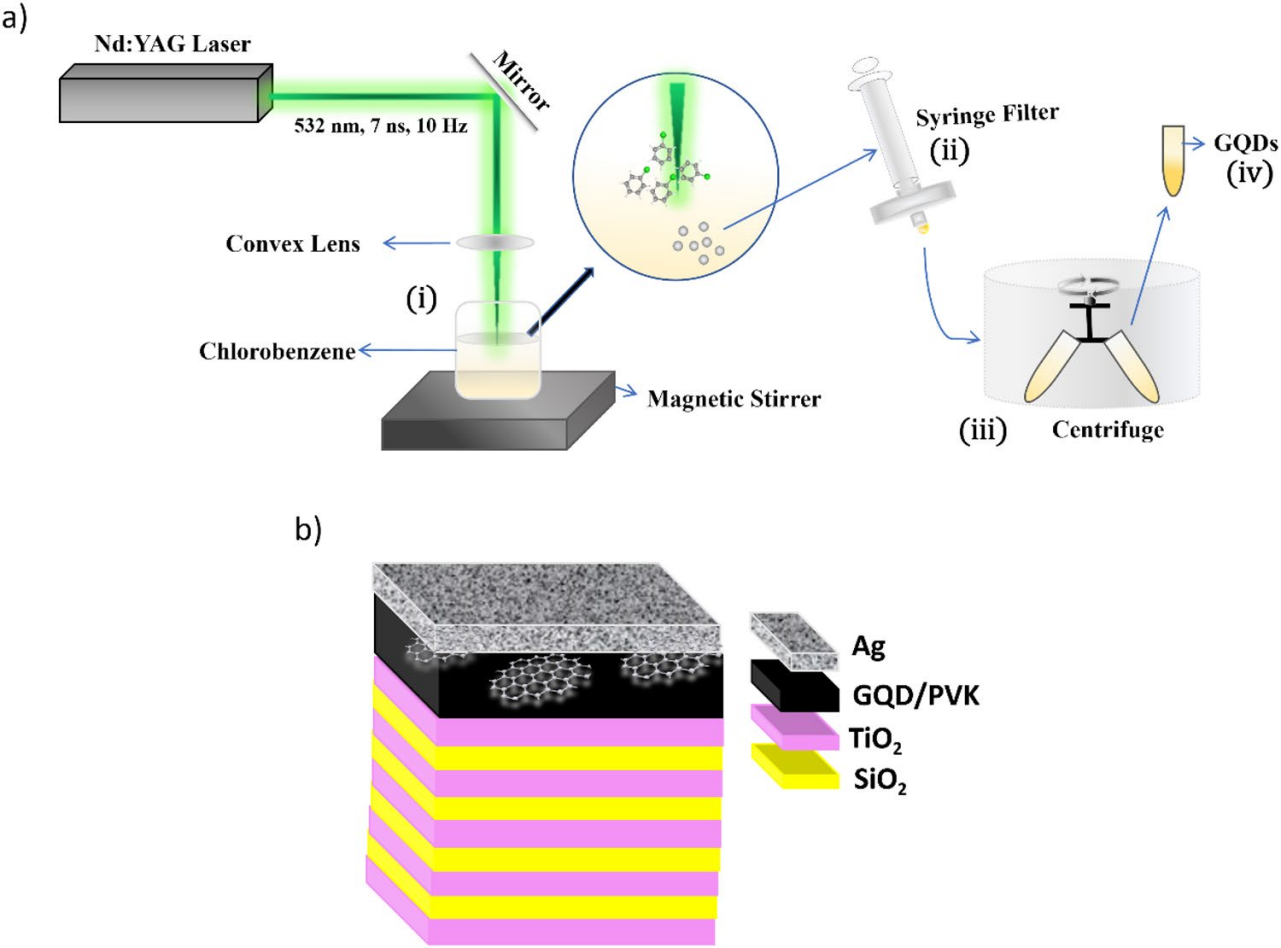
Schematic of the GQD formation by PLIL (i) PLIL with a Q-switched Nd:YAG laser (532 nm wavelength, 7 ns pulse width and 10 Hz repetition rate) in chlorobenzene along with stirring the liquid, (ii) filtration by a syringe filter, (iii) centrifugation done at 6000 rpm for 20 min and (iv) GQDs and b) the fabricated TPC structure with 4.5 bilayers of TiO_2_ and SiO_2_, a spacer layer of GQDs incorporated in PVK and a 35 nm thick Ag film.

The PLIL technique is a straightforward method for creating nanoparticles in liquids^43^. It has numerous intriguing advantages over other nanoparticle production techniques, including high purity, size, and shape control, scalability, adaptability, cost efficiency, and an environmentally friendly nature, as it does not create any byproducts or requires toxic reducing agents^44^. Using the PLIL technique, we synthesized GQDs in chlorobenzene (Fig. 1a). The excitation source was a Q-switched Nd:YAG laser with 532 nm wavelength, a pulse width of 7 ns, a repetition rate of 10 Hz, and a pulse energy of 30 mJ. During the irradiation process, the laser beam was tightly focused to the center of 5 ml of chlorobenzene taken in a 10 ml glass beaker using a convex lens with a focal length of 10 cm. The duration of irradiation was 8 min. When a laser beam is focused on a liquid, it rapidly heats and vaporizes a small portion of the liquid, which then expands rapidly and creates a plasma plume^45,46^. This reaction may lead to the formation of fragments of carbon chains. These fragments can then react with each other to form a graphitic network. The increased surface energy of these graphitic networks in solution can cause them to curl around a structural defect, resulting in the formation of the observed GQDs^47^.The colour change from transparent to brownish indicated the formation of GQDs. We used a 0.45 microfilter to purify the GQD solution. Then, the solution was centrifuged at 6000 rpm for 20 min. The supernatant containing GQDs was then collected for the study.

The sol–gel technique is a versatile method for producing nanoparticles with tailored properties and functionalities^48,49^ as well as uniform thin films of metal oxides^50^. It is a simple and cost-effective method that can be carried out at low temperatures and is particularly useful for synthesizing heat-sensitive materials. We used the sol–gel method to synthesize TiO_2_ and SiO_2_ for the fabrication of DBR. The detailed synthesis process is given in the supporting information. In brief, titanium butoxide, methanol, and Glacial Acetic Acid (GAA) were used at a volume ratio of 0.3:5:0.42 and vigorously mixed by a magnetic stirrer to make a TiO_2_ sol. Tetraethyl orthosilicate, ethanol, and GAA were used at a volume ratio of 0.37:5:0.57 to make a SiO_2_ sol, followed by vigorous stirring.

### Fabrication of photonic structures

The spin coating technique is a popular and widely used technique for the deposition of thin films due to its advantages of uniformity, reproducibility, speed, cost-effectiveness, and versatility^51,52^. Using sequential dynamic spin coating of TiO_2_ and SiO_2_ on a clean glass substrate at predetermined rotation speeds and times, a DBR structure starting with TiO_2_ and having 4.5 bilayers was fabricated. All the films were deposited at a quarter-wave thickness to maximize the reflectance. Each layer was annealed at 110 °C to form a uniform film. We utilized a polymer called Poly (9-vinyl carbazole) (PVK) dissolved in chlorobenzene to insert the defect material GQD as a spacer layer because we are producing GQDs in this solvent. To improve the optical quality of the structure, it was again calcined at 110 °C. Silver of precalibrated thickness (35 nm) was deposited by the thermal evaporation method to create the TPC structure. Silver is advantageous over gold, aluminum, and other metals for Tamm plasmon structures due to its high-quality factor, minimal absorption coefficient, and absence of interband transitions within the visible spectrum, resulting in reduced optical losses in this range^53^. A schematic of the TPC structure is given in Fig. 1b.

### Characterization

We used a Jeol/JEM 2100 High-Resolution Transmission Electron Microscope (HRTEM) to confirm the formation of graphene as quantum dots. The creation of GQDs was verified by analysing the absorption spectra of the materials at wavelengths ranging from 250 to 900 nm using a UV–visible spectrometer (Shimadzu-UV 2450). The Raman spectrum of the synthesized GQDs was obtained using a LabRAM HR Evolution Raman Spectrometer. To study the Fourier transform infrared (FTIR) spectra of the samples, a Perkin Elmer Spectrum Two spectrometer was used. We optimized the thickness of the TiO_2_ and SiO_2_ thin films by measuring the reflectance of the coated films using a Shimadzu-UV 2450 spectrometer. Scanning electron microscopy of the fabricated structure was performed with a Gemini 300 Scanning Electron Microscope (SEM). To examine the NLO properties, we employed a Q-switched Nd:YAG laser with a 532 nm wavelength, a 7 ns pulse width, and a 10 Hz repetition rate. We were able to examine the structure's optical limiting (OL) characteristics by plotting the normalized transmittance values against the input fluence. We used a Horiba Fluoromax-4 spectrophotometer to examine the fluorescence, quantum yield, and lifetime properties of the synthesized GQDs and fabricated structures. For theoretical studies, we used Transfer Matrix simulations in MATLAB, and the electric field distribution inside the structure was studied using COMSOL Multiphysics 6.0.

## Results and discussion

### Properties of GQDs

**Figure 2:**
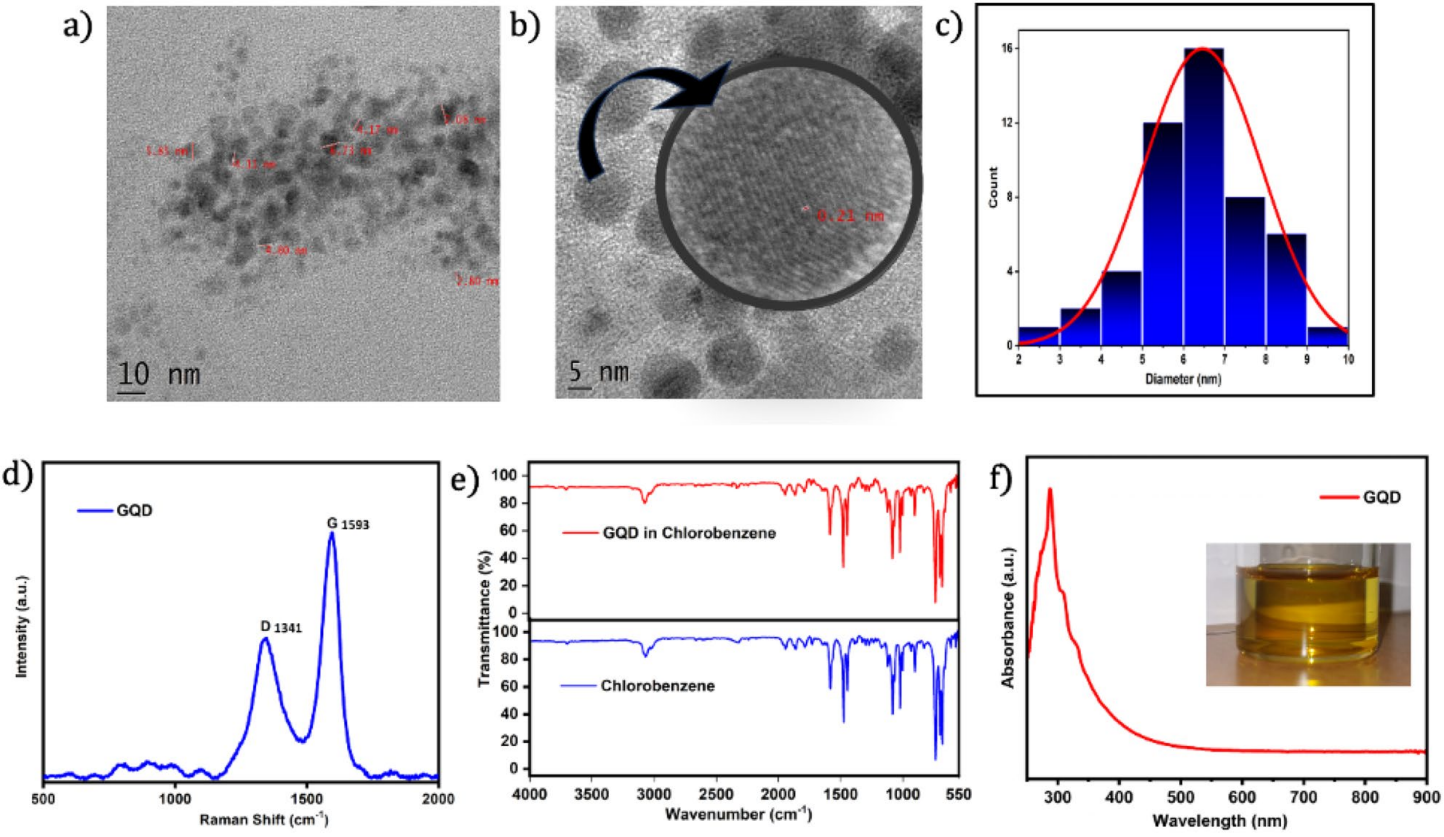
(**a**, **b**) HRTEM images of GQDs in chlorobenzene (inset of (**b**) shows the magnified image of a single quantum dot with an interplanar spacing of 0.21 nm indicating the (100) plane of the crystalline structure, (**c**) particle size distribution histogram showing an average size of 6.5 nm, (**d**) Raman spectrum of GQDs with the D and G bands, (**e**) the comparison of FTIR spectra of colloidal GQDs and pure chlorobenzene indicating the lack of functional changes after laser irradiation and (**f**) absorption spectrum of GQDs in chlorobenzene with a characteristic peak at approximately 286 nm (inset shows the appearance of colloidal GQDs under visible light).

TEM images of GQDs are shown in Fig. 2a,b. This confirmed the formation of GQDs with an average size distribution of 6.5 nm (Fig. 2c). Figure 2b shows that the interplanar distance in GQDs is 0.21 nm, which indicates the presence of the (100) plane in their crystalline structure^30^.

The Raman spectrum of the synthesized GQDs (Fig. 2d) consists of two prominent peaks. One peak found at 1593 cm^-1^ is the G band (graphene band), which corresponds to the E_2g_ vibrational mode of sp^2^-hybridized carbon atoms in graphene lattice structures. The G band is a property of graphitic materials connected with carbon‒carbon bond in-plane stretching. Another peak at 1341 cm^-1^ is the D band, which is caused by structural flaws or disorders in the graphene lattice. This corresponds to the breathing mode of carbon atoms in the sp^2^ and sp^3^ hybridization states. The I_D_/I_G_ value, commonly known as the Raman disorder parameter, is a quantitative measure used to characterize structural disorder in carbon-based materials such as graphene and graphene-related materials. The I_D_/I_G_ value is typically close to zero or very small for pristine graphene since the D band is usually weak or absent in high-quality graphene samples^47^. However, when defects are introduced, such as in GQDs, the I_D_/I_G_ increases, indicating the presence of structural disorder. The I_D_/I_G_ value we obtained is 0.57, which suggests a moderate level of ordered graphitic structure and defect density.

The FTIR spectrum of colloidal GQDs (Fig. 2e) is similar to that of chlorobenzene, revealing that the solvent after laser irradiation is still chlorobenzene^54^. Figure 2f shows the UV‒visible absorption spectrum of the prepared GQDs, which has a sharp peak at 286 nm, and the absence of significant absorption in the visible range (400–700 nm) makes them an efficient candidate for incorporation as a defect material in a photonic crystal for NLO studies.

**Figure 3:**
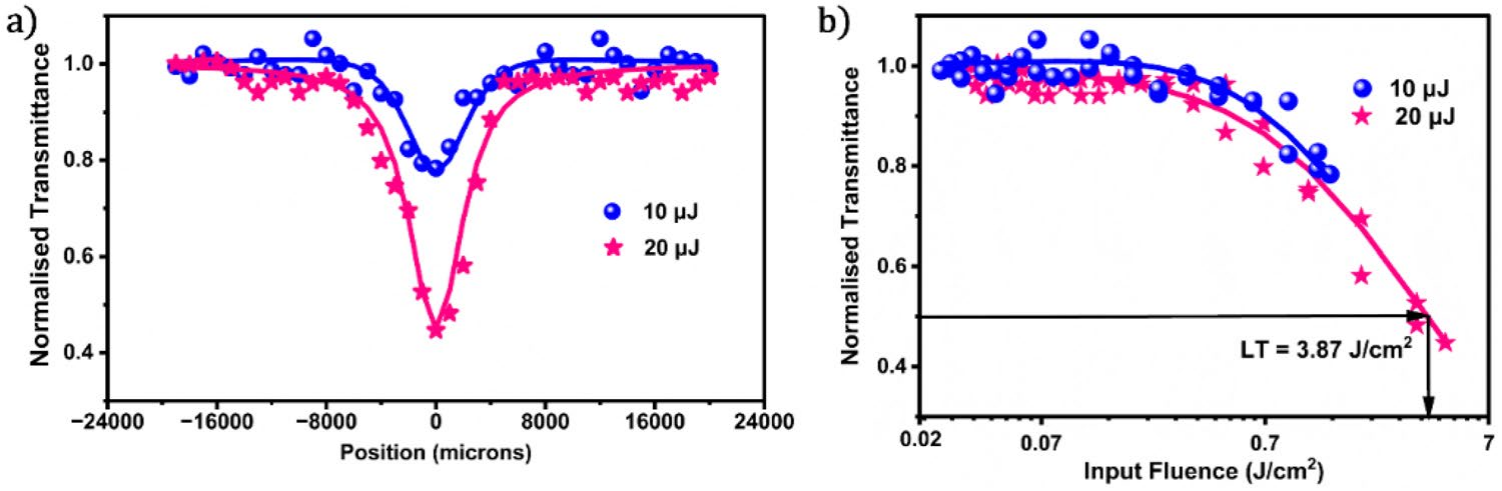
(**a**) Z-scan profiles of the sample at different energies and (**b**) corresponding optical limiting characteristic plots showing an optical limiting threshold of 3.87 J/cm^2^ for the GQDs.

Nonlinear optical studies of the colloidal GQDs at different on-axis intensities are shown in Fig. 3a. The GQDs exhibit significant Reverse Saturable Absorption (RSA) behaviour at the observed energies. Generally, an RSA-like profile is observed due to any of the processes like Excited state Absorption (ESA), Two-Photon Absorption (2PA), Free Carrier Absorption (FCA), etc., or merged effects of any of these processes^55^. When the excitation wave lacks enough energy to excite the electron from its ground state by a single photon, TPA is typically observed. In this case, the simultaneous absorption of two photons at a high input intensity via a middle virtual level yields a nonlinear optical response. It is clear from the UV‒visible absorption spectrum of the synthesized GQDs in chlorobenzene (Fig. 2f) that there is only weak absorption corresponding to the excitation wavelength (532 nm). In light of this, it is possible that in our sample, the higher absorption and transmittance dip is caused by the increased likelihood of 2PA occurring at higher intensities. Nonlinear scattering was not substantial when measured with a detector 45 degrees normal to the sample.

The theoretical model that best matches the experimentally observed data, from which *I*_*s*_, the saturation intensity, and *β*_*eff*_, the effective nonlinear absorption (NLA) coefficient, are derived, may be found using the nonlinear pulse propagation equation given by

1 $$\frac{{dI}}{{dz\prime }} = - \left( {\frac{{\alpha _{0} }}{{1 + I/I_{S} }} + \beta _{{eff}} I} \right)I$$

Here, I stand for incident intensity, *dI/dz’* represents the shift in intensity across the material thickness, and α_0_ is the linear absorption coefficient. We obtained *β*_*eff*_ values close to 25 cm/GW for both energies, which in turn strengthens our proposed reason for RSA being 2PA^56^.

The optical limiting capacity of the GQDs was then examined using the data directly from the open aperture z-scan results. The nonlinear optical phenomenon known as optical limiting occurs when the incident light intensity increases above a certain threshold. This behavior is particularly useful in applications where protection against intense light pulses, such as laser beams, is needed^57^. The ideal optical limiter is extremely transparent at low light intensities but becomes opaque at higher light intensities^58^. It has a larger damage threshold and a lower onset value and limiting threshold (LT). LT is the value of the input fluence corresponding to half of the linear transmittance. Figure 3b shows the optical limiting plots of GQDs. We obtained an LT value of 3.87 J/cm^2^.

**Figure 4:**
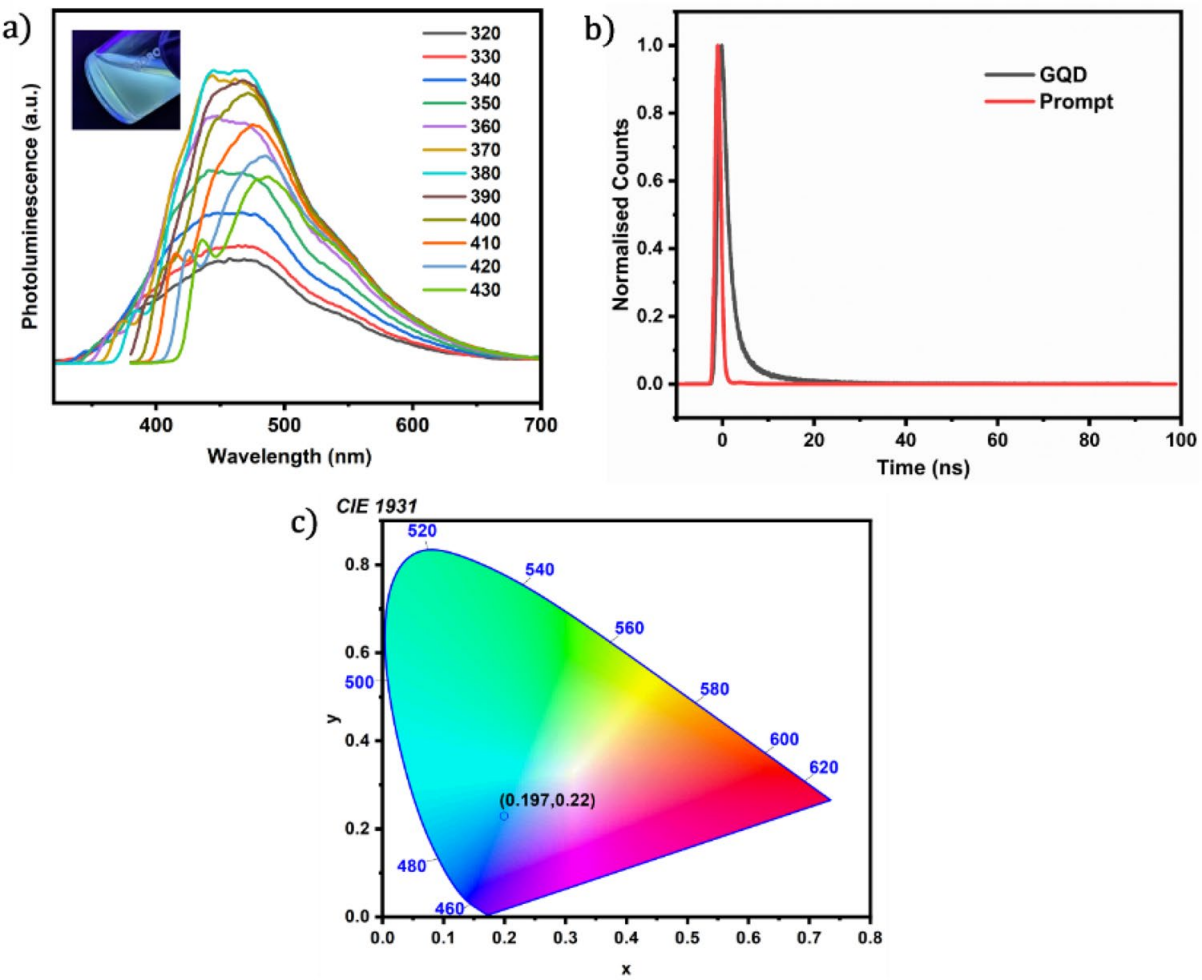
(**a**) Photoluminescence spectra of GQDs in chlorobenzene at different excitation wavelengths ranging from 320 to 430 nm (the inset shows the fluorescence of GQDs under UV light), (**b**) the fluorescence decay profile of the synthesized GQDs and the prompt and (**c**) CIE chromaticity diagram for GQDs showing the scope for blue-emitting LEDs.

Photoluminescence (PL) studies are useful for determining the optical characteristics of materials^59,60^. The synthesized GQDs had a moderate value of absolute quantum yield which is about 19.4 ± 0.165. The PL spectra of the synthesized GQDs at several excitation wavelengths spanning from 320 to 430 nm are shown in Fig. 4a. It is obvious that GQD is a good fluorescent material with a broadband emission spectrum in the near-UV and visible regions, and the maximum emission is obtained for an excitation wavelength of 380 nm. The fluorescence of the synthesized GQDs is shown in the inset of Fig. 4a. Lifetime measurements of the as-synthesized GQDs were taken by the Time-Correlated Single Photon Counting (TCSPC) technique (Fig. 4b). The decay of GQDs can be thought of as a combination of three underlying processes since a 3-exponential function is required to fit the obtained profile. The obtained time constants (T) and relative amplitudes (A) are given in Table 1. The equation given below was used to find the mean lifetime of the sample, and the calculated mean lifetime at an excitation wavelength of 380 nm was $\tau$ =3.44 ns.

**Table 1:** The time constants and relative amplitudes obtained for the fluorescence decay curves of GQDs.

	Time constant, T (ns)	Norm. amplitude, A
1	5.09	0.182
2	1.38	0.71
3	14.34	0.11

2 $${\tau =A}_{1}{T}_{1}+{A}_{2}{T}_{2}+{A}_{3}{T}_{3}$$

The CIE 1931 xy chromaticity diagram, also known as the CIE chromaticity diagram, is a graphical representation of the colors visible to the human eye. It was developed by the Commission Internationale de l'Éclairage (CIE), also known as the International Commission on Illumination^61^. The diagram is a two-dimensional plot with axes labelled x and y, and it represents all the chromaticities that the human eye can perceive. The colour purity of an LED is determined by the position of the x and y coordinates of the LED along this line. The obtained values were (x, y) = (0.197, 0.22). The findings suggest that GQDs hold significant promise as materials suitable for optical devices. These materials could serve as viable options for blue LEDs powered by UV chips, finding applications across various advanced technologies.

Till now, we have been discussing the synthesis and characterization of GQDs. The results show that the GQDs synthesized by laser irradiation in chlorobenzene have exceptional properties such as stable photoluminescence, chemical stability, good optical limiting capability, etc. These results are comparable with the properties of GQDs produced by the PLIL method in another benzene derivative solvent such as toluene^30,42^.

### Properties of TPC

**Figure 5:**
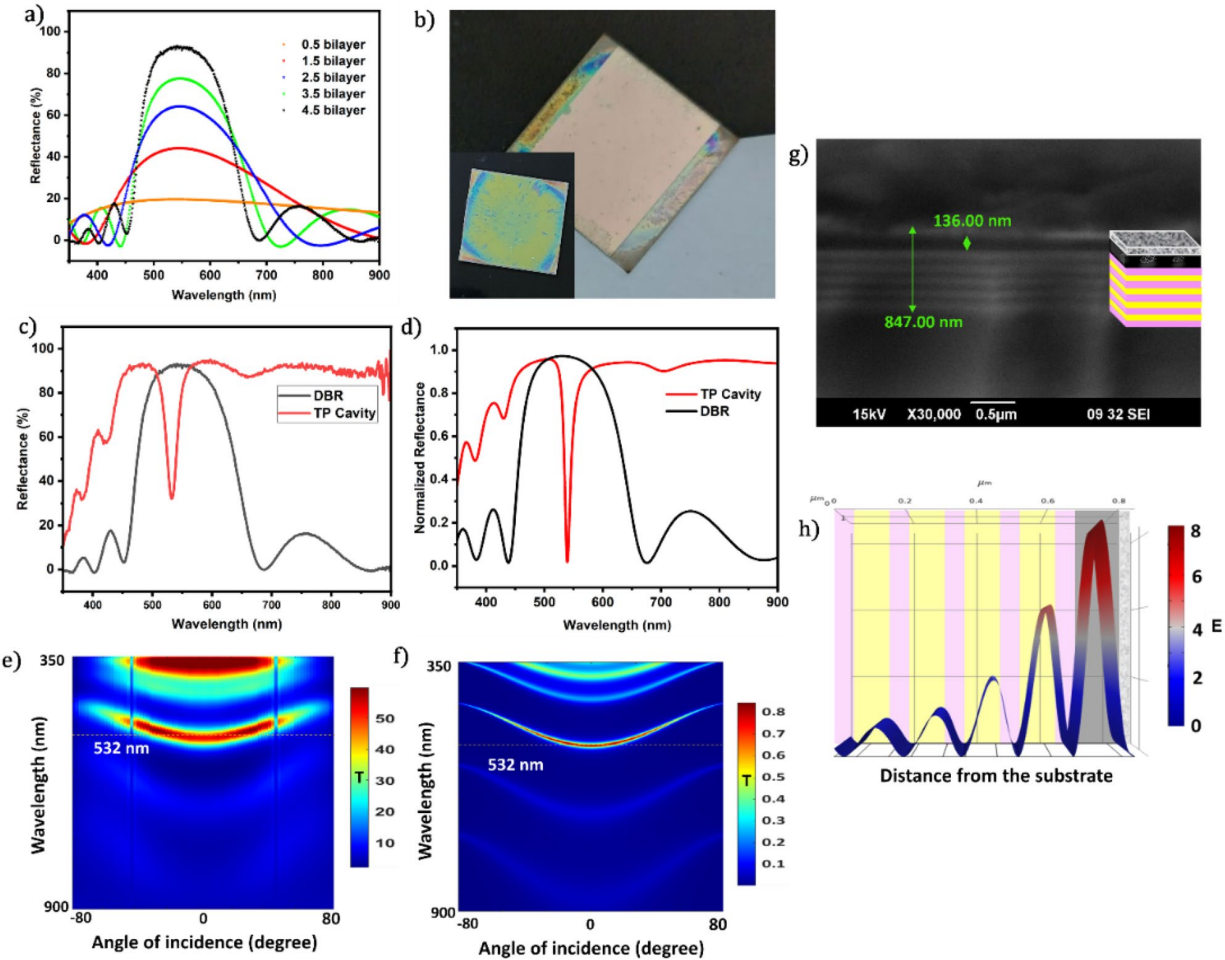
(**a**) Reflectance spectra of the DBR for different numbers of bilayers starting from the TiO_2_ layer, (**b**) photograph of the fabricated TPC structure (inset is a photograph of the DBR), (**c**) comparison between the obtained reflectance spectra of the DBR and TPC, (**d**) comparison between the simulated reflectance spectra of the DBR and TPC, (**e**) angular dispersion of the transmittance of the fabricated TPC structure showing the transmittance mode, (**f**) simulated angular dispersion of the transmittance of the TPC structure, (**g**) cross-sectional SEM image of the TPC structure where each layer aligns with the layers of the structure, as depicted in the inset, and h) simulated electric field distribution in the TPC structure at 532 nm, showing a sevenfold enhancement in the electric field within the TP cavity.

To investigate how the structure affects the optical limiting characteristics and fluorescence of GQDs, we have now integrated the synthesized GQDs into a TPC structure. The detailed results of the fabrication of the TPC structure are given in Fig. 5. The reflectance spectra for different bilayers of the fabricated DBR using TiO_2_ and SiO_2_ as alternative layers are shown in Fig. 5a. As the number of bilayers increases, the reflectance increases along with the narrowing of the peak. We obtained a reflectance peak of 91% centered at 542 nm for 4.5 bilayers and a bandwidth of 178 nm. Photographic images of the synthesized TPC structure and DBR are given in Fig. 5b. The reflectance profiles of the fabricated DBR and TPC (Fig. 5c) are in good agreement with the simulated spectra (Fig. 5d) for the structures, which indicates the quality of the fabricated TPC structure. The obtained TP mode was at 539 nm with 32% reflectance. It has a better profile compared to similar works^12,62,63^.

To determine the quality of the TP mode, we studied the angular dispersion characteristics of the transmittance of the fabricated TPC (Fig. 5e). The transmittance of light through a material varies based on the angle at which the light reaches the surface, which is affected by the refractive characteristics of the material. The angular dispersion of the fabricated TPC structure shows that the TPC mode matches that simulated (Fig. 5f) for the structure, which again confirms the quality of our TPC structure. The ability to adjust the transmission mode by changing the angles of incidence indicates that this structure has the potential to serve as a bandpass filter with tunable transmittance. The cross-sectional SEM image of the TPC structure (Fig. 5g) shows the uniformity of the layers of the structure. The simulated electric field distribution of the TPC structure is given in Fig. 5h, which can provide valuable insights into the behaviour of the TPC structure, such as the position and intensity of the electric field. This indicates a sevenfold field enhancement at the spacer layer, which in turn enhances the light-matter interaction.

**Figure 6:**
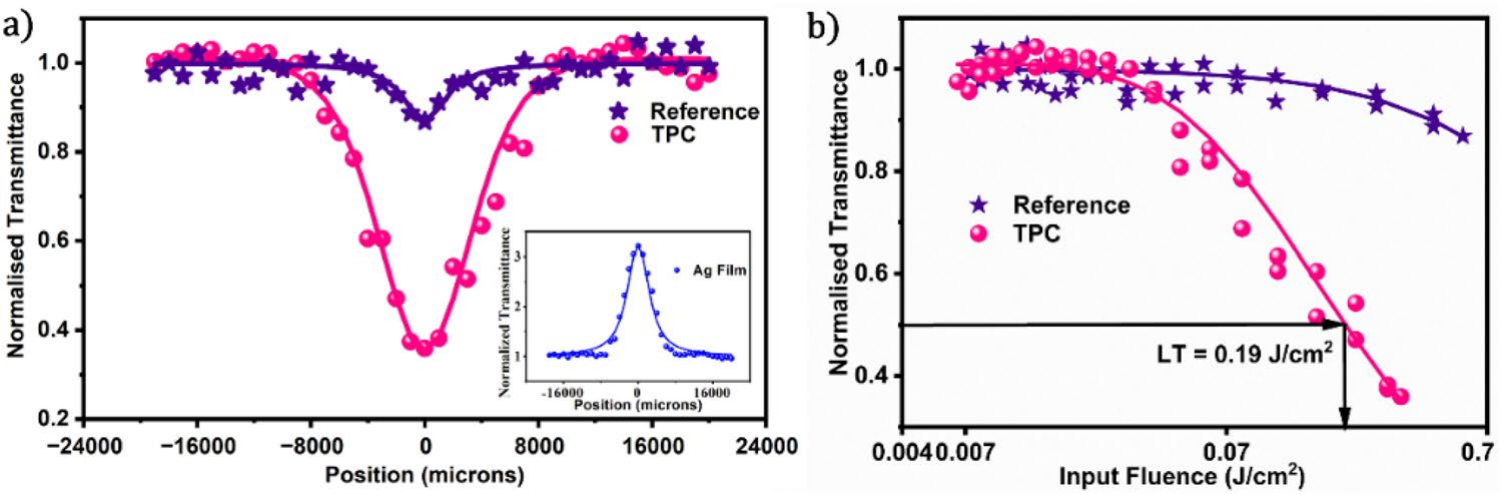
(**a**) Comparison of the OA Z-scan plots of the TPC and the GQD/PVK reference film (the inset shows the SA characteristics of a 35 nm Ag film) at 0.07 GW/cm^2^ on-axis intensity, and (**b**) corresponding OL plots showing the limiting threshold value of the TPC structure as 0.19 J/cm^2^.

Improving optical nonlinearity can lead to the development of more efficient and advanced optical systems, resulting in better performance and capabilities in a variety of applications. We used OA Z-scan analysis to determine the optical nonlinearity enhancement of the TPC structure in comparison to that of the reference film. Since the TP mode was centered at 539 nm in our structure, we had to tune the TP mode to the excitation wavelength (532 nm) for the NLO studies. Thus, the TPC film was kept at an angle of 18° concerning normal to the incoming laser beam. Figure 6a depicts the nonlinear absorption characteristics of the reference and TPC structures at 0.07 GW/cm^2^ on-axis intensity. They exhibit RSA behaviour, with the steeper valley of the TPC structure ensuring a significant increase in optical nonlinearity. A 35 nm Ag film showed SA at 0.07 GW/cm^2^ on-axis intensity as shown in the inset of Fig. 6a. SA is a phenomenon where a material's absorption capacity reduces as the input light intensity rises which in turn increases the transmittance during the process. The theoretical best fit of the profiles was obtained using Eq. (1), and the obtained data are given in Table 2. By comparing the data obtained for the TPC and reference film, we obtain a 43-fold increase in the nonlinear absorption coefficient. To the best of our knowledge, our structure has the best-obtained enhancement factor compared to previous research that used Tamm plasmon structures to boost optical nonlinearity (Table 2).

**Table 2:** Comparison of the effective nonlinear absorption coefficient, limiting threshold, and NLA enhancement factor of various photonic crystal cavity structures.

Structure	Cavity Material	*β*_*eff*_* (*10^5^ cm/GW)	LT (J/cm^2^)	Enhancement Factor in NLA
Reference†	GQD/PVK	1.5	–	–
Tamm [TiO_2_/SiO_2_(4.5): Ag]†	GQD/PVK	65	0.19	43
Tamm [CA/PVK (10): Au]^12^	ZnP+ /CA	0.89	1.04	6
Tamm [TiO_2_/SiO_2_(4.5): Au]^62^	PVK/Au@C	34	–	38
Tamm [PVK/CA (9.5): Au]^63^	Bodipy/CA	2.08	–	5.6
Cavity [TiO_2_/SiO_2_(10.5)]^64^	ZnO	0.26	0.74	4
Cavity [PVK/CA (10)]^65^	Pt_17_	0.36	1.007	4.5
Cavity [SiO_2_/TiO_2_(9)]^66^	BaTiO_3_	0.58	–	7

^†^This work.

Through data analysis of OA z-scan studies, we examined the OL capabilities of the TPC structure. The TPC structure demonstrated optical limiting behaviour with a 0.017 J/cm^2^ limiting onset and a 0.19 J/cm^2^ limiting threshold, as shown in Fig. 6b. These values, which we consider to be either greater than or on par with previously published optical limiting thresholds of analogous photonic structures, as stated in Table 2, demonstrate the ability of our structure to perform as an efficient optical limiter. This implies that our structure can improve the performance, safety, and efficiency of different optical systems and devices. Moreover, the ability to control and manipulate these nonlinear interactions holds significant promise for the development of advanced photonic devices, including those such as all-optical switches and optical modulators.

Photoluminescence investigations of the TPC structure revealed a substantial departure from the PL spectrum of the GQD/PVK reference film (Fig. S1a in the supporting information). We obtained the maximum emission of the GQD/PVK reference film when excited at 346 nm rather than at 380 nm. As a result, we performed PL investigations on every film sample using an excitation wavelength of 346 nm. Notably, the spectral profile of the reference film is different from that of colloidal GQDs, which are characterized by wide PL emission. By comparison, the reference film has a narrower emission profile that is blue shifted, and its maximum emission occurs at 433 nm when excited at a wavelength of 346 nm. This discrepancy can be attributed to various factors, including the physical shape of the material, molecular arrangement, orientation, surface effects, interactions with PVK, and other elements that collectively modify the photoluminescence spectrum. These variations can impact both the wavelength and intensity of light emitted by the material, thus influencing the observed photoluminescence spectrum.

The excitation spectrum of the GQD/PVK reference film for emission peaks at approximately 433 nm is given in the supporting information (Fig. S1b). However, the emission spectra of the TPC exhibit intriguing behavior, as shown in Figure S1c in the supporting information. A tunable emission spectrum is observed with changes in the angle of incidence of the excitation wave. When we increase the angle of incidence from 0° to 70°, the emission peak shifts towards shorter wavelengths, accompanied by a notable increase in the emission intensity. This wavelength shift aligns with the changes observed in the transmittance spectra of the TPC as a function of the angle of incidence, as shown in Figure S1d.

**Figure 7:**
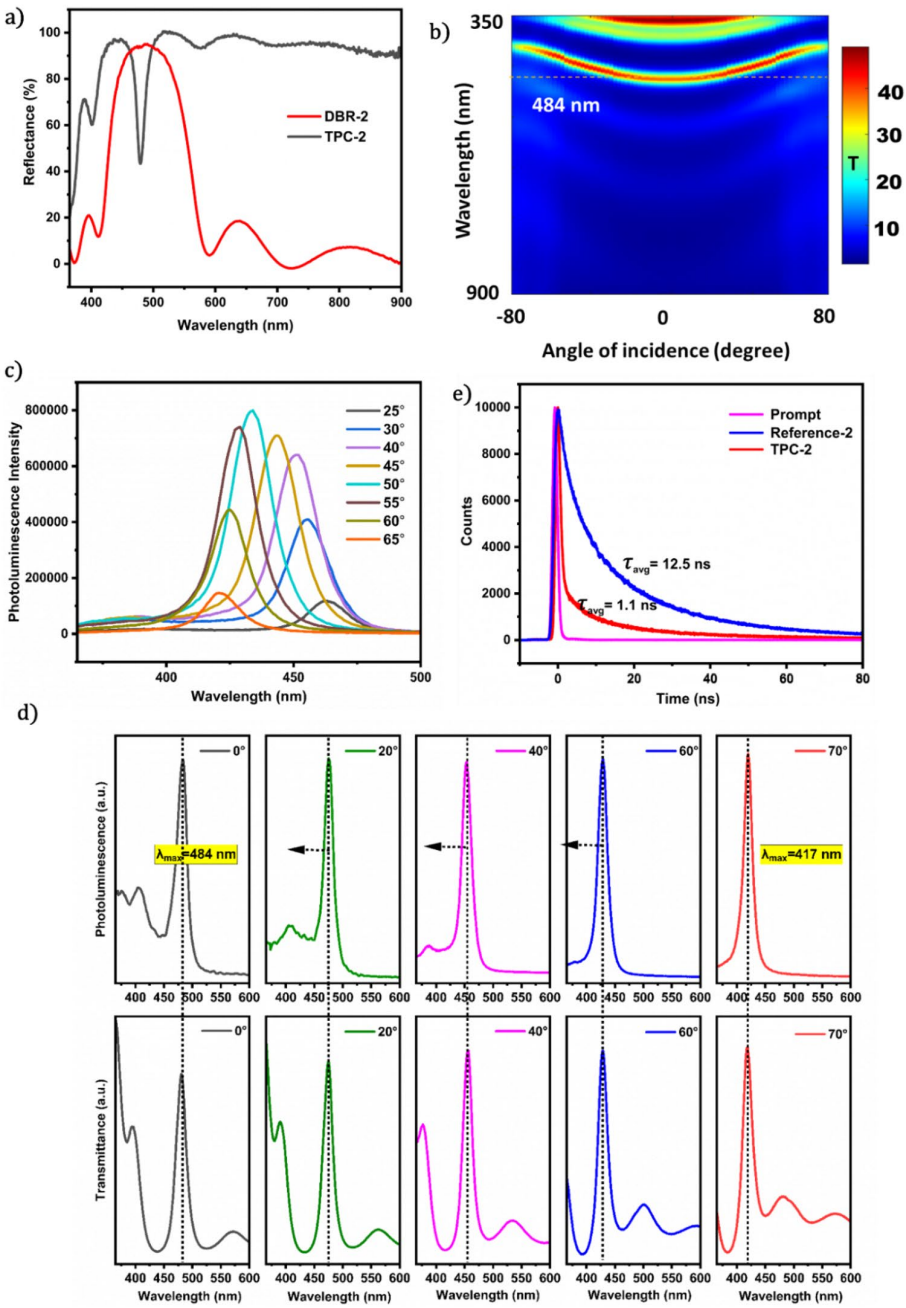
(**a**) A comparison between the obtained reflectance spectra of DBR-2 and TPC-2 showing the TPC mode inside the bandgap of DBR-2, (**b**) angular dispersion of the transmittance of the TPC-2 structure showing the angle tunable transmittance mode, (**c**) PL spectra of the TPC-2 structure for different angles of incidence at 346 nm excitation wavelength, (**d**) a comparison of the obtained emission profile of TPC-2 with its transmittance spectra for different angles of incidence showing the tuning of fluorescence aligned with the transmittance mode shift from 484 to 417 nm and (**e**) a comparison of the fluorescence decay profile of reference-2 and TPC-2 showing the lifetime quenching of TPC-2 (the decay profile of the prompt is also shown).

We designed our TPC structure to have a cavity around 532 nm to satisfy the resonance condition for studying the nonlinear optical behaviour. We obtained very interesting results for the PL studies of the TPC (Fig. S1d in the supporting information), even though it has a cavity mode far from the intrinsic emission peak of the GQD/PVK reference film. Therefore, to study the PL properties, we purposefully designed another TPC structure (TPC-2) with a cavity closer to the maximum emission of the GQD/PVK reference film. The details of the fabrication are given in the supporting information in Sect. 1b. The results for the TPC-2 structure are given in Fig. 7. We constructed the TPC mode at approximately 484 nm to explore the tunability of the emission around the intrinsic emission maxima of the GQD/PVK reference film (Fig. 7a). The angular dispersion of the transmittance (Fig. 7b) indicates the tunability of the transmittance mode with respect to the angle.

The PL emission profiles of TPC-2 at different angles of incidence are shown in Fig. 7c. Here, we can observe a blueshift and enhancement in the emission peak with increasing incidence angle. However, beyond the inherent maximum peak position, i.e., 433 nm, increasing the angle decreases the PL intensity. This can be attributed to the fact that the bare film itself shows maximum emission at a wavelength of approximately 433 nm. As shown in Fig. 7d, TPC-2 exhibits unique PL spectra with a very distinct peak that can be tuned from 484 to 417 nm by solely changing the angle of incidence from 0 to 70 degrees.

In TPC or similar structures, the confined wavelength corresponds to the resonance wavelength at which light is effectively trapped or confined within the structure. Changing the angle of incidence (excitation angle) changes the optical path length and thus the resonance condition, leading to a shift in the confined wave. The maximum photoluminescence intensity is observed when the emission wavelength and resonance mode coincide.

The photonic bandgap and TPC play key roles in the observed tuning of the fluorescence of GQDs^67^. A photonic bandgap refers to a specific frequency range with no available electromagnetic modes, and no light propagation is allowed. Within this frequency range, the spontaneous emission of light is entirely suppressed^68^. The primary reason for the enhancement of fluorescence in the TPC structure can be attributed to the Purcell effect^9^. The Purcell effect is a phenomenon in which the spontaneous emission rate of a fluorescent emitter is modified when it is placed within a resonant optical cavity^69^. The local density of optical states is altered within the Tamm plasmon cavity. This indicates that the fluorophores have more accessible photon states for emission. As a result, the spontaneous emission rate of the fluorophores increases. In simple terms, they release photons faster than they would in free space. The enhanced emission rate, facilitated by the Purcell effect, leads to an increase in the radiative decay rate of the fluorophores. Consequently, more of the excited fluorophores undergo radiative decay and emit photons, which results in higher fluorescence intensity at the resonance wavelength.

The Purcell factor (F) is a measure of the enhancement of the spontaneous emission rate of a quantum emitter in the presence of a cavity, and it is defined as the ratio of the emission rate (or radiative decay rate) in the cavity (${\Gamma }_{c}$) to the emission rate in free space (${\Gamma }_{0}$)^70,71^. Mathematically, the Purcell factor is given by:

3 $$F=\frac{{\Gamma }_{c}}{{\Gamma }_{0}}$$

The radiative decay rates in the cavity and free space can be expressed as ${\Gamma }_{0}=\frac{1}{{\uptau }_{0}}$, and ${\Gamma }_{c}=\frac{1}{{\uptau }_{c}}$, where ${\uptau }_{0}$ is the radiative lifetime of the emitter in free space and ${\uptau }_{c}$ is the radiative lifetime of the emitter when coupled to the cavity.

4 $$\therefore F=\frac{{\uptau }_{0}}{{\tau }_{c}}$$

Therefore, to determine the Purcell factor, we obtained information about the radiative lifetimes of the emitter both when it was coupled to the cavity (TPC-2) and in free space (i.e., Reference-2). These values were calculated from the TCSPS data obtained for the structures, as shown in Fig. 7e. The details of the lifetime calculations for these structures are given in the supporting information Sect. 2. The average lifetime obtained for the reference film was ${\uptau }_{0}$=12.5 ns, whereas for TPC-2, it was very short, i.e., ${\uptau }_{c}$=1.1 ns. Therefore, we obtained an experimental Purcell factor, F = 11.36, by using Eq. (5), which is comparable to previously reported values^71^.

Thus, by strategically placing GQDs within the Tamm cavity structure, we can significantly tune and amplify the emission intensity and collection efficiency of emitted photons. For instance, when we are looking into the sensing application of GQDs, the broad emission spectrum of GQDs spans a wide range of wavelengths, which makes them useful for detecting and sensing various substances. However, at low concentrations of substances, changes in the wavelength or intensity of emission may be difficult to identify. This is where the incorporation of GQDs into TPC structures becomes crucial. As illustrated in Fig. S2 in the supporting information, integrating GQDs with TPC enhances the emission profile, resulting in increased intensity (Figure S2a) and sharper emission peaks (Figure S2b). This improvement allows for the detection of small variations in emission wavelength and intensity, providing better sensitivity to the presence of trace elements in different materials. This heightened sensitivity is particularly crucial in medical diagnostics, environmental monitoring, and industrial sensing. TPC can amplify fluorescence peak shifts caused by the introduction of biomolecules, making it a reliable indicator of their presence^72^. Moreover, fluorescence tunability allows precise control of emission properties, facilitating multiplexed sensing and reducing spectral overlap in complex samples. The ability to tailor the fluorescence characteristics of probes enhances selectivity and enables the design of sensors optimized for specific applications. These advancements collectively contribute to improved accuracy, increased versatility, and expanded applicability of fluorescence-based sensing technologies, making them invaluable tools in scientific research, healthcare, and environmental management.

An advantage of this method is the flexibility of tailoring GQDs synthesized through various methods to suit the specific requirements of different applications. For example, using GQDs produced in water instead of chlorobenzene enhances biocompatibility, making them better suited for biosensing applications^73^. This adaptability allows researchers and engineers to optimize the properties of GQDs, such as size, shape, and functionalization, for maximum performance within the TPC structures.

This combination allows for precise tuning of optical properties, resulting in improved light-matter interactions and a stronger Purcell effect, which leads to enhanced fluorescence and nonlinear optical responses. Additionally, the compact and efficient nature of TPCs makes them suitable for integration into miniaturized devices and applications. Although the integration of GQDs with TP structures offers exciting potential for different applications, there are some limitations and thresholds in terms of the enhancement effect and scalability for practical applications. There are challenges in scaling up the production of TPCs while maintaining precise geometries and uniformity across larger areas, which may increase production costs. Material selection must account for compatibility with GQDs and other system components, considering properties such as the refractive index, dielectric constant, and thermal stability. Additionally, the design of DBRs within TPC structures, including the number of bilayers, influences performance and scalability. While increasing the number of bilayers can improve the quality factor and light confinement, it also increases the complexity of the fabrication process and may affect the overall device size. The Purcell effect is dependent on the quality factor and mode volume of the TPCs, which may vary and impact the degree of enhancement. Thermal effects from high laser intensities can affect stability and performance, requiring effective thermal management.

Despite these challenges, careful optimization and ongoing research can facilitate practical solutions, such as refining material compatibility and enhancing mode matching for maximum enhancement. Efficient thermal management and reduced photon loss will ensure consistent performance and long-term stability, paving the way for scalable and reliable TPC/GQD-based technologies.

## Conclusion

GQDs were synthesized by a facile laser irradiation method, and the ability of TPC structures to improve their optical properties was explored. GQDs have shown promising characteristics for diverse applications. They exhibited notable nonlinear optical behavior attributed to TPA, making them potential candidates for optical-limiting applications. Additionally, they displayed efficient fluorescence and favourable chromaticity, indicating their suitability for use in advanced technologies, particularly in the development of blue LEDs. The incorporation of GQDs into a TPC structure resulted in a 43-fold increase in the nonlinear absorption coefficient due to enhanced light-matter interactions. Optical limiting studies of the structure showed a limiting threshold value of 0.19 J/cm^2^, demonstrating the structure's efficiency in acting as an optical limiter. Photoluminescence studies showed tunable and enhanced emission spectra in the TPC structures attributed to the Purcell effect, with a calculated Purcell factor of 11.36. The angular dispersion characteristics of the transmittance of the TPC structure indicated that the structure operates as an angle-tunable frequency filter. The findings highlight the potential of tailoring TPC structures integrated with GQDs for improved performance in various fields, from optical technologies to biomedicine, especially in advanced sensing applications, emphasizing their enhanced optical limiting properties and tunable fluorescence characteristics.

### Supplementary Information

Supplementary Information.

## Data Availability

Data are available from the corresponding author on reasonable request.
