# Supplementary material for: Impact of Tamm plasmon structures on fluorescence and optical nonlinearity of graphene quantum dots
Source: Sci Rep. 2024 Jun 10;14:13255. doi: 10.1038/s41598-024-62694-9 (PMC11164873; doi:10.1038/s41598-024-62694-9)
Supplement: Supplementary file 1 — Supplementary Information. [file 41598_2024_62694_MOESM1_ESM.pdf]

<u>**SUPPORTING INFORMATION**</u>

Impact of Tamm Plasmon Structures on Fluorescence and Optical Nonlinearity of Graphene Quantum Dots

Hasana Jahan Elamkulavan, Nikhil Puthiya Purayil, Sanjay Subramaniam and Chandrasekharan Keloth*
Laser and Nonlinear Optics Laboratory, Department of Physics, National Institute of Technology, Calicut- 673601, India
*E-mail address: csk@nitc.ac.in

1. Synthesis Method

a. Synthesis of TiO_2 and SiO_2

To make TiO_2 nanoparticles, titanium alkoxide precursor (Titanium Butoxide (TBOT)) is added drop-by-drop to a mixture of methanol and Glacial Acetic Acid (GAA) in a volume ratio 0.3: 5:0.42 and vigorously mixed by a magnetic stirrer to make a TiO_2 sol. To make SiO_2 nanoparticles, similarly add a silicon alkoxide precursor (Tetra Ethyl Ortho Silicate (TEOS)) to a mixture of ethanol and GAA in a volume ratio of 0.37:5:0.57 to make a SiO_2 sol followed by vigorous stirring. The sol of TiO_2 and SiO_2 are then aged to allow their nanoparticles to expand and aggregate, generating a gel. After that, the gel is coated on a glass substrate which can be dried and calcined to eliminate any remaining solvent and generate the TiO_2 and SiO_2 thin films.

b. Synthesis of TPC-2

A DBR structure with 5.5 bilayers of TiO_2 and SiO_2 was fabricated using the spin coating method. All films were deposited at a quarter-wave thickness to enhance reflectance at 484 nm wavelength. Each layer was annealed at 110 °C to form a uniform film of the required nanoparticles. GQD in PVK dissolved in chlorobenzene was incorporated as the defect material. 35 nm of silver was deposited by the physical vapor deposition method to create the Tamm Plasmon structure.

2. Results and Discussion

a. Photoluminescence spectra of GQD

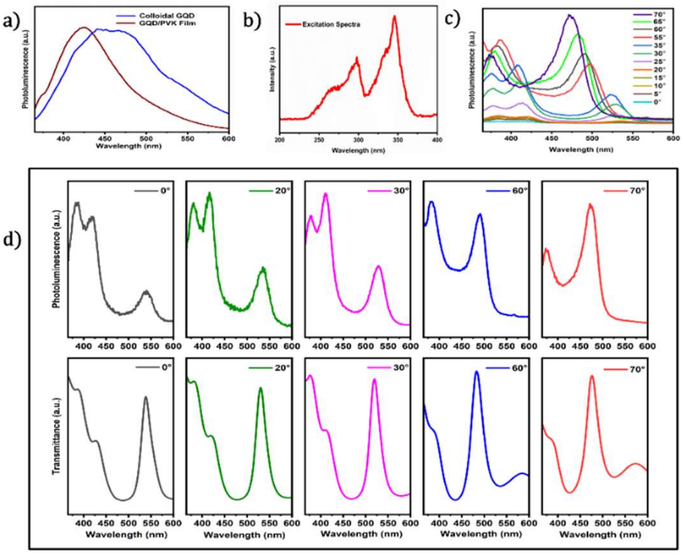

Figure S1: a) A comparison of the PL spectra of colloidal GQD and GQD/PVK reference film at an excitation wavelength of 346 nm, b) excitation spectrum of GQD/ PVK reference film for the emission peak around 433 nm, c) the PL spectra of TPC structure for different angles of incidence at 346 nm excitation, d) a Comparison of the obtained emission profile of TPC with transmittance spectra for different angles of incidence

b. Lifetime Studies of TPC-2 and Reference-2

Table T2: Time constants and normalized amplitudes obtained for the lifetime measurement of TPC-2 and Reference-2 by using the TCSPC technique

**TPC-2**	**Time constant, T (ns)**	**Norm. Amplitude, A**
**1**	0.047	0.02
**2**	0.97	0.97
**3**	11.02	0.018
**Reference-2**	**Time constant, T (ns)**	**Norm. Amplitude, A**
**1**	7.02	0.33
**2**	24.04	0.43
**3**	1.892	0.144

c. A comparison of PL from GQD/PVK reference film (Reference-2) and TPC-2

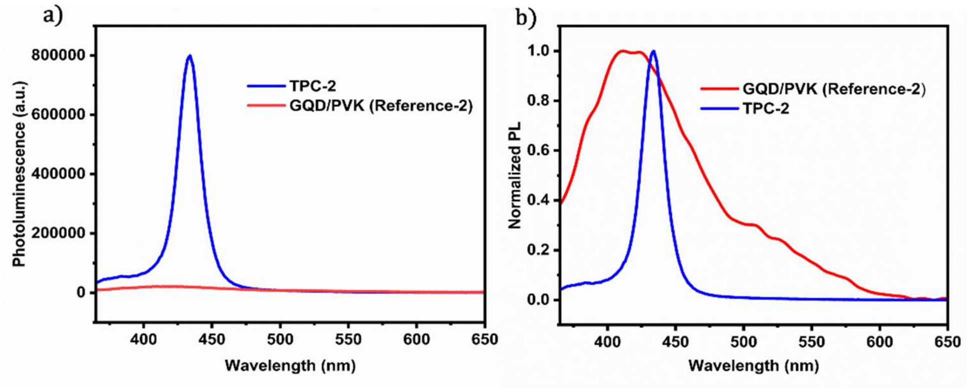

Figure S*2*: a) a) A comparison of the emission spectrum of the GQD/PVK reference film and TPC-2 (at an angle of incidence of 50°) showing the intensity enhancement when incorporating the GQD within the spacer layer and b) a comparison of the normalized emission spectrum of the GQD/PVK reference film and TPC-2 (at an angle of incidence of 50°) showing the narrowed emission peak when incorporated the GQD withing the spacer layer
